# Exploring Supramolecular Assembly Space of Cationic 1,2,4-Selenodiazoles: Effect of the Substituent at the Carbon Atom and Anions

**DOI:** 10.3390/molecules27031029

**Published:** 2022-02-02

**Authors:** Mariya V. Grudova, Alexey S. Kubasov, Victor N. Khrustalev, Alexander S. Novikov, Andreii S. Kritchenkov, Valentine G. Nenajdenko, Alexander V. Borisov, Alexander G. Tskhovrebov

**Affiliations:** 1 Peoples’ Friendship University of Russia, Miklukho-Maklaya Street 6, 117198 Moscow, Russia; shokoi@mail.ru (M.V.G.); vnkhrustalev@gmail.com (V.N.K.); platinist@mail.ru (A.S.K.); 2 Kurnakov Institute of General and Inorganic Chemistry, Russian Academy of Sciences, Leninsky Prosp. 31, 119071 Moscow, Russia; fobosax@mail.ru; 3 N.D. Zelinsky Institute of Organic Chemistry, Russian Academy of Sciences, Leninsky Prosp. 47, 119334 Moscow, Russia; 4 Saint Petersburg State University, Universitetskaya Nab. 7/9, 199034 Saint Petersburg, Russia; a.s.novikov@spbu.ru; 5 Lomonosov Moscow State University, Leninskie Gory 1/3, 119991 Moscow, Russia; nenajdenko@gmail.com; 6 R.E. Alekseev Nizhny Novgorod State Technical University, Minin St. 24, 603155 Nizhny Novgorod, Russia; avb1955@rambler.ru; 7 N.N. Semenov Federal Research Center for Chemical Physics, Russian Academy of Sciences, Ul. Kosygina 4, 119991 Moscow, Russia

**Keywords:** selenodiazoles, non-covalent interactions, chalcogen bonding, cyclization reactions, cycloaddition, nitriles, selenium heterocycles

## Abstract

Chalcogenodiazoles have been intensively studied in recent years in the context of their supramolecular chemistry. In contrast, the newly discovered cationic 1,2,4-selenodiazole supramolecular building blocks, which can be obtained via coupling between 2-pyridylselenyl halides and nitriles, are virtually unexplored. A significant advantage of the latter is their facile structural tunability via the variation of nitriles, which could allow a fine tuning of their self-assembly in the solid state. Here, we explore the influence of the substituent (which derives from the nitrile) and counterions on the supramolecular assembly of cationic 1,2,4-selenodiazoles via chalcogen bonding.

## 1. Introduction

Recent decades have seen significant progress in the creation of artificial supramolecular systems with increasing complexity. Hydrogen bonding (HB) and coordination at the metal center has been employed often in the creation of complex structures from simpler building blocks [1,2,3,4]. Although the construction of larger and more complex structures is an important task within this field, the other major challenges include the utilization of other weak interactions (apart of HB and metal coordination) for obtaining more complex aggregates, determining recognition units, as well as designing and synthesizing novel recognition units. The attractive non-covalent interactions between polarizable main group elements and Lewis basic species, which include chalcogen bonding (ChB) and halogen bonding (XB), have recently attracted considerable attention and emerged as powerful alternatives to complexation and HB in supramolecular chemistry [5,6,7,8,9,10,11,12].

The progress in ChB chemistry includes broad applications in organocatalysis, molecular recognition, anion transport, self-assembly, etc. [13,14,15,16]. Chalcogenodiazoles have gained particular interest within ChB research and have been extensively studied in recent years in the context of their applications in anion recognition and as supramolecular building blocks [6,17,18,19,20,21].

We recently introduced novel cationic 1,2,4-selenodiazole building blocks, which can be easily prepared via the coupling between 2-pyridylselenyl halides and nitriles in high yields [22,23]. In contrast to the exhaustively studied earlier chalcogenodiazoles, the supramolecular chemistry of these novel 1,2,4-selenodiazole is virtually unexplored, but could be a fruitful research field in the near future. A significant advantage of novel 1,2,4-selenodiazole building blocks is their facile structural tunability via the variation of nitriles, which could allow the fine tuning of their self-assembly in the solid state. Here, we explore the effect of the substituent (which derives from the nitrile) on the supramolecular assembly of cationic 1,2,4-selenodiazoles via ChB non-covalent interactions.

## 2. Results and Discussion

**Scheme 1:**
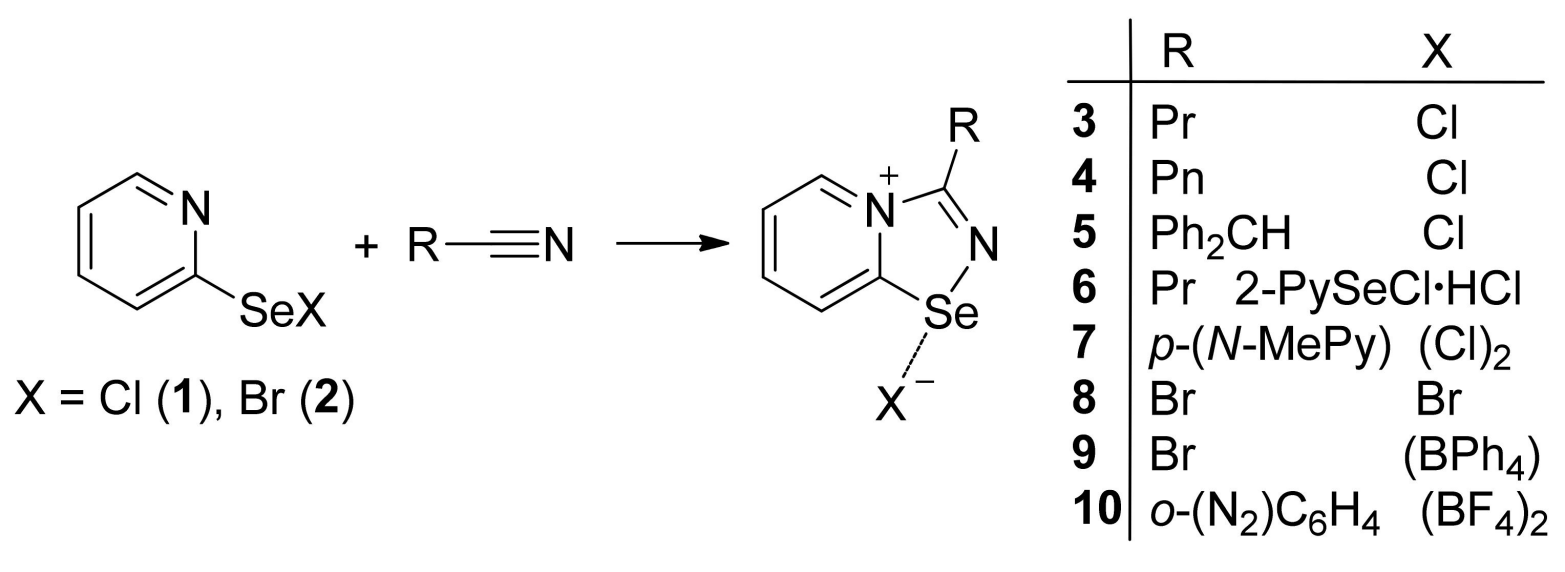
Synthesis of **3**–**10**. For the conditions, see the Materials and Methods.

Cationic 1,2,4-selenadiazoles **3**–**10** studied within the framework of the current work were synthesized in high yields, according to our method, with small variations when necessary (Scheme 1, Experimental Section).

**Figure 1:**
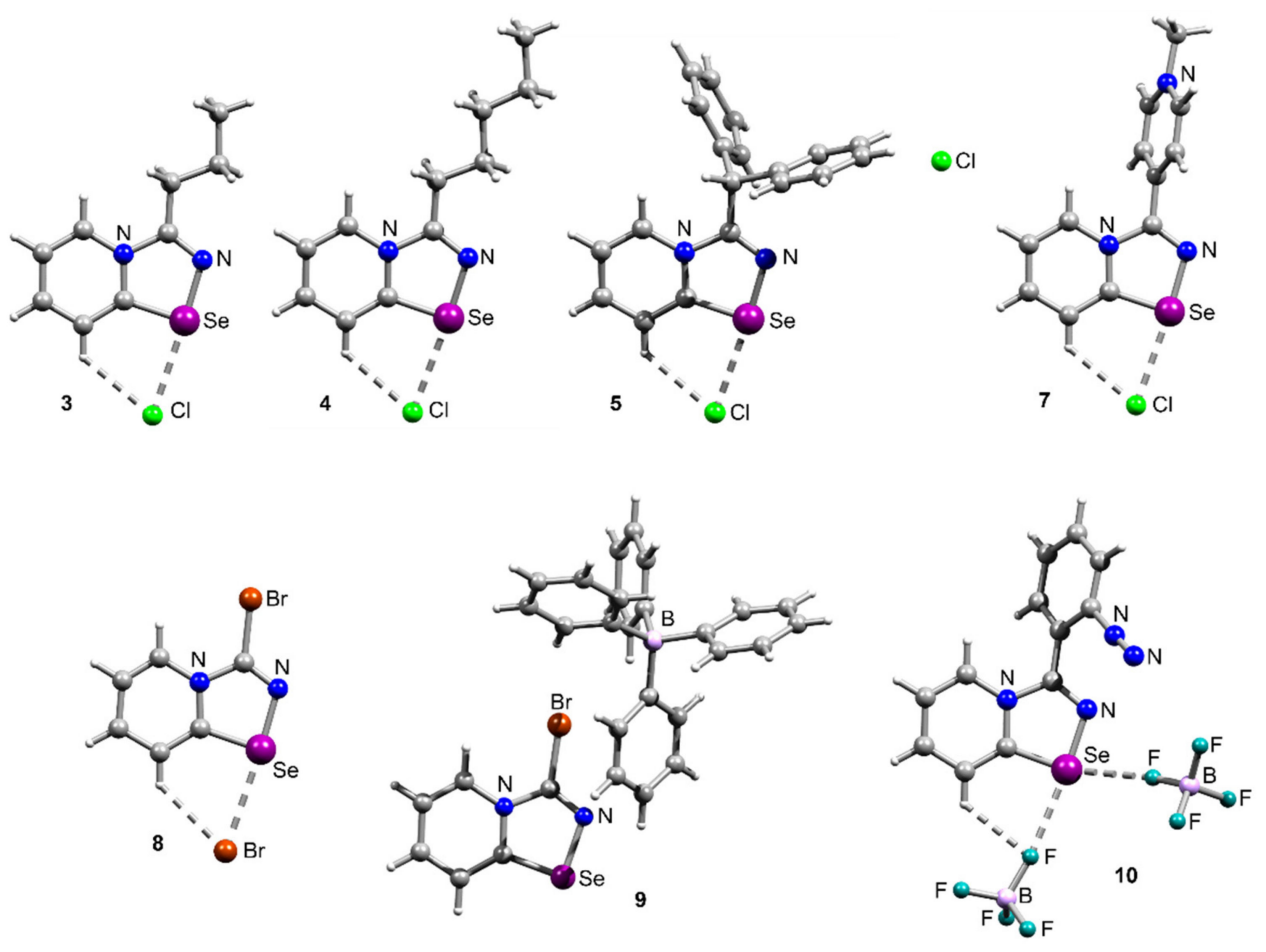
Ball-and-stick representations of the crystal structures of **3**–**5** and **7**–**10**. Grey and light-grey spheres represent carbon and hydrogen, respectively.

^1^H and ^13^C{^1^H} NMR spectra in D_2_O confirmed the formation of cyclic 1,2,4-selenadiazoles **3**–**10**. The adducts **3**–**10** readily recrystallized from dichloromethane or methanol to give single crystals suitable for analysis by single-crystal X-ray crystallography, which confirmed the formation of cationic 1,2,4-selenadiazoles (Figure 1). The N–Se distance for **3**–**10** was in the interval between 1.824(3) Å and 1.860(4) Å, and was typical for the N–Se single bond. The C=N separation (1.25(1)–1.291(5) Å for **3**–**10**) corresponded to a typical double bond [24,25,26,27,28,29,30,31,32,33].

**Figure 2:**
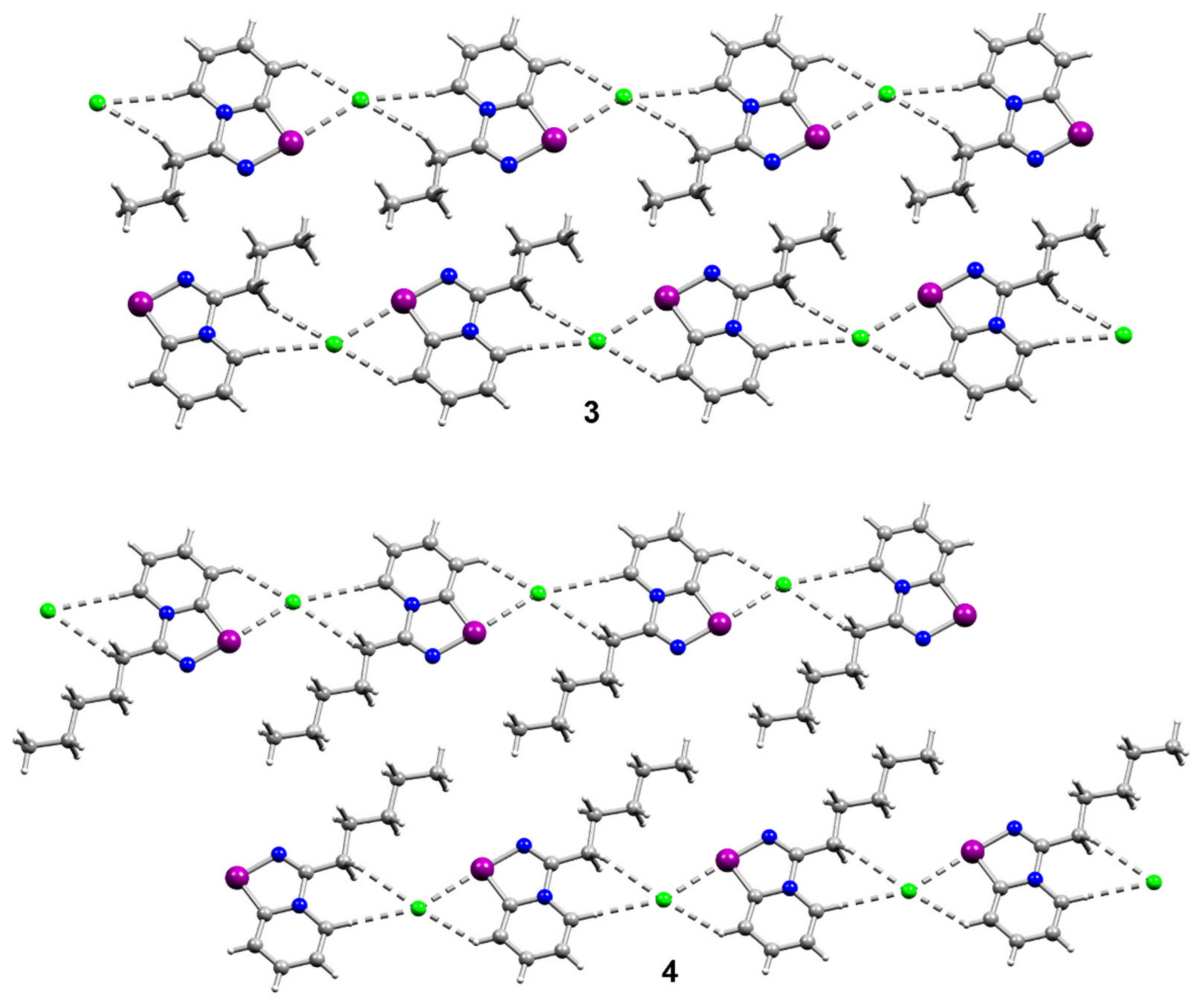
Ball-and-stick representations of the crystal structures of **3** and **4**, demonstrating their self-assembly into 1-D supramolecular polymers via Se∙∙∙Cl and H∙∙∙Cl interactions. Green, purple, blue, grey, and light-grey spheres represent chlorine, selenium, nitrogen, carbon, and hydrogen, respectively.

Earlier, we established that the adduct derived from propionitrile formed [Se–Cl]_2_ squares in the solid state. Interestingly, when we switched higher homologs (i.e., *n*-butyronitrile and even *n*-hexanenitrile), the corresponding adducts **3** and **4** did not form dimers in the solid state, but self-assembled into 1-D supramolecular polymers via Se∙∙∙Cl ChB and H∙∙∙Cl HB (Figure 2). In these cases, other weak interactions outcompeted [Se–Cl]_2_ and [Se–N]_2_ square formations in the solid state. Expectedly, the transoid position towards the N atom around the selenium center was occupied by the chloride due to the presence of the supporting H∙∙∙Cl HB, but the cisoid position remained unoccupied in the crystals of **3** and **4**.

**Figure 3:**
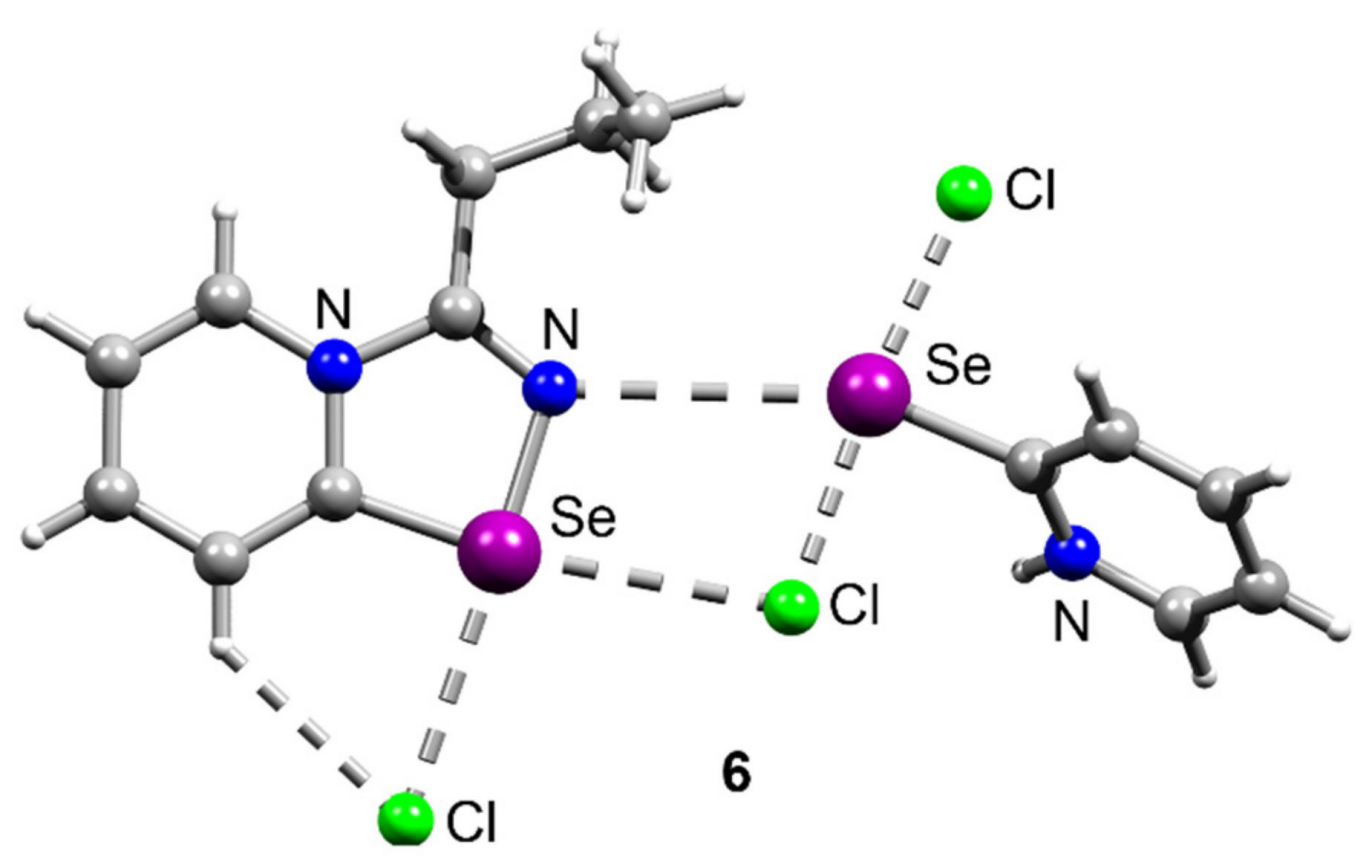
Ball-and-stick representation of the crystal structure of **6**, demonstrating attractive Se∙∙∙N, Se∙∙∙Cl, and H∙∙∙Cl interactions. Grey and light-grey spheres represent carbon and hydrogen, respectively.

When the solution of **3** was slowly crystallized from dichloromethane over a period of ca. one month, aside from the major product **3**, a minor amount of crystals of **6** also precipitated (Figure 3). 

Compound **6** represented an adduct of 1,2,4-selenadiazole **3** with 2-pyridylselenylchloride hydrochloride. The heterodimer **6** featured Se–N–Se–Cl squares, which we have not observed previously for the adducts of nitriles with 2-pyridylselenylhalides. Compound **6** could potentially form via partial hydrolysis of **3**, which results in the formation of HCl, and could react with another molecule of **3** to generate 2-PySeCl HCl. The latter could coprecipitate with an additional molecule of **3** to form **6**. However, further investigations are required for detailed insight of unusual heterodimers containing Se–N–Se–Cl squares.

**Figure 4:**
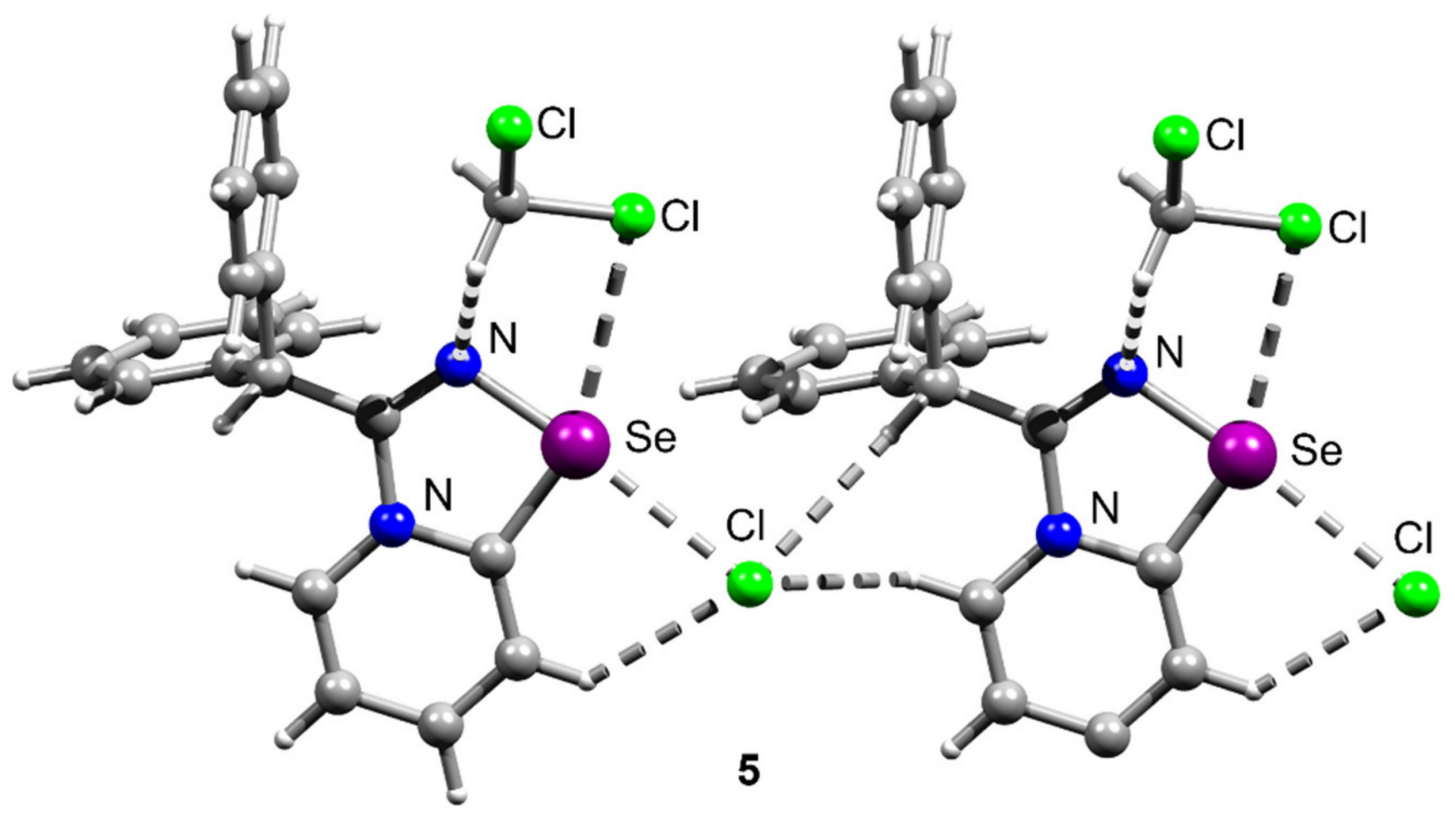
Ball-and-stick representation of the crystal structure of **5**, demonstrating its self-assembly into 1-D supramolecular polymer via Se∙∙∙Cl and H∙∙∙Cl interactions and ChB interactions with a cocrystallized CH_2_Cl_2_ molecule. Green, purple, blue, grey, and light-grey spheres represent chlorine, selenium, nitrogen, carbon, and hydrogen, respectively.

Further we switched to a bulkier diphenylacetonitrile, which formed an expected adduct **5** with 2-pyridylselenylchloride and self-assembled into 1-D chains via Se∙∙∙Cl and H∙∙∙Cl interactions (Figure 4) in the solid state, in the same fashion as **3** and **4**. However, in contrast to **3** and **4**, adduct **5** contained a cocrystallized CH_2_Cl_2_ molecule which featured a ChB interaction with a selenium center and occupied a cisoid position at the selenium atom (Figure 4). Arguably, this was allowed by the pocket formed by the bulky diphenylmethyl substituent.

**Figure 5:**
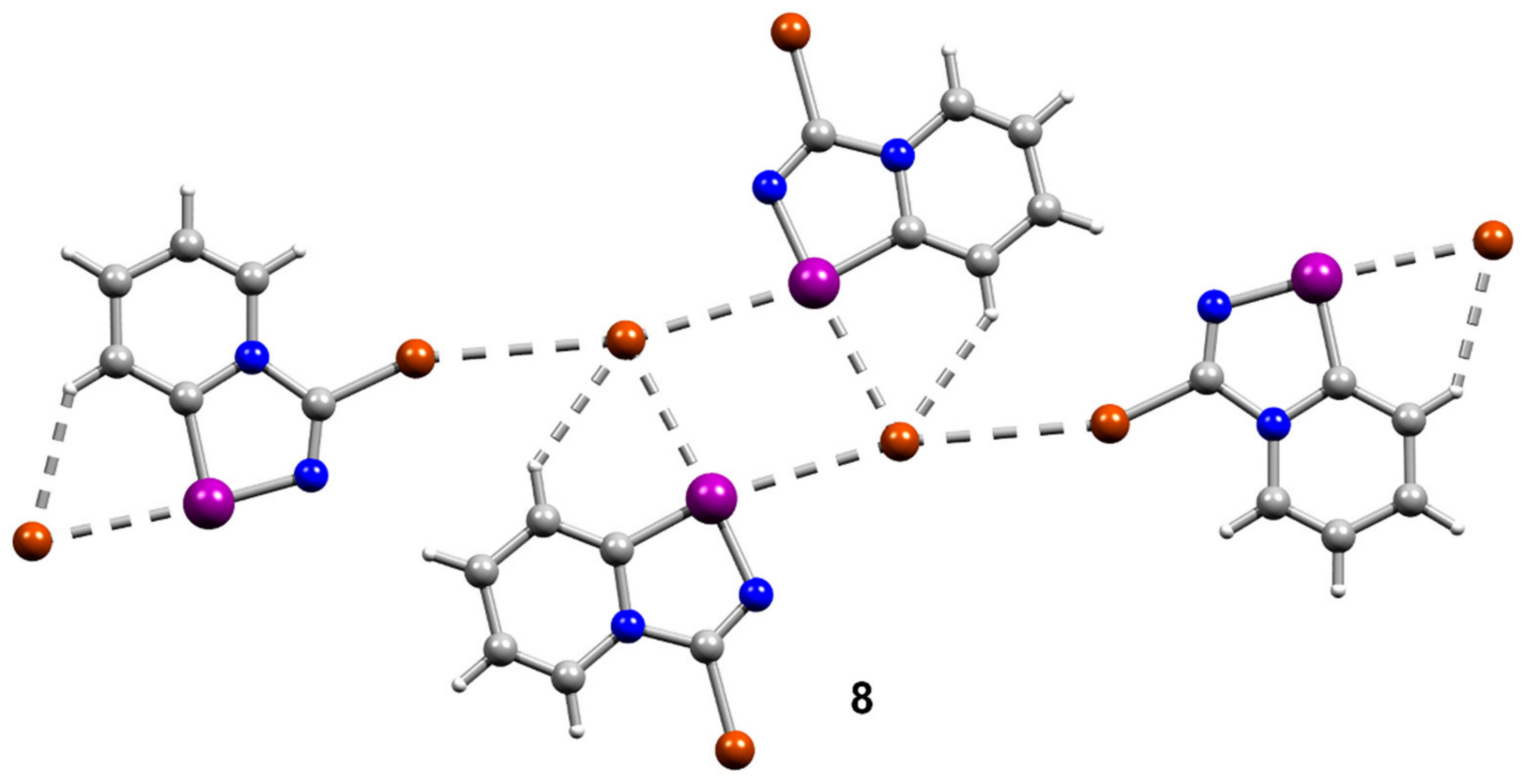
Ball-and-stick representation of the crystal structure of **8**, demonstrating its self-assembly into 1-D supramolecular polymer via Se∙∙∙Br and Br∙∙∙Br interactions. Brown, purple, blue, grey, and light-grey spheres represent bromine, selenium, nitrogen, carbon, and hydrogen, respectively.

Previously, we showed that 2-pyridylselenylchloride eagerly reacts with cyanogen bromide forming a corresponding cationic 1,2,4-selenadiazole in an almost quantitative yield [23]. Within this work, we explored the reaction of 2-pyridylselenylbromide with cyanogen bromide, which also resulted in the formation of the adduct **8**, which self-assembled in the solid state in the same manner as its chloride analog, which featured [Se–Cl]_2_ square dimers in the solid state (Figure 5). Interestingly, switching from a chloride to a bromide resulted in an intriguing peculiarity: the Br(C)–Br distance (3.678 Å) in the crystal of **8** was shorter than the Br(C)–Cl separation (3.774 Å) in the analogous adduct of 2-pyridylselenylchloride with cyanogen bromide [23]. This indirectly indicates that the Br∙∙∙Br interactions in **8** are attractive. Overall, molecules of **8** formed 1-D polymer via Se∙∙∙Br ChB and Br∙∙∙Br XB in the crystal.

**Figure 6:**
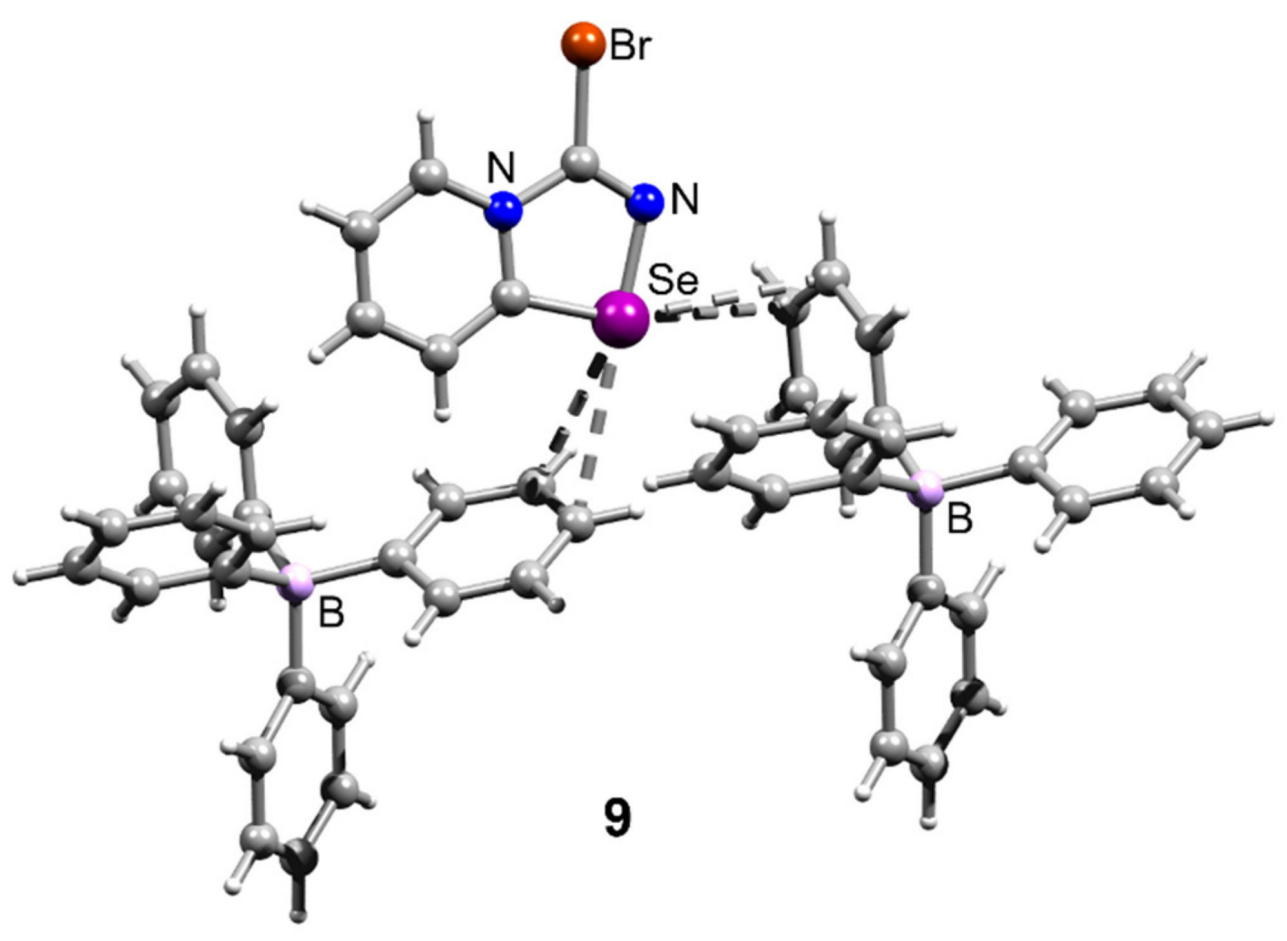
Ball-and-stick representation of the crystal structure of **9**, demonstrating attractive Se∙∙∙π interactions between the selenadiazole cation and two neighboring BPh_4_ anions. Grey and light-grey spheres represent carbon and hydrogen, respectively.

Further, we were interested in how variation of the anion would affect the self-assembly of the adduct 2-pyridylselenylchloride with cyanogen bromide. For this reason, we performed an addition of a saturated solution of NaBPh_4_ in MeOH to a solution of **8** in the same solvent, which resulted in an immediate formation of microcrystalline precipitate of **9** (Figure 6). 

Introduction of the bulky anion resulted in the destruction of the polymeric chain. The solid-state structure of **9** featured “isolated” 1,2,4-selenadiazole cations which were involved in two chalcogen–π interactions. It should be noted that chalcogen–π interactions are a bonding motif found in biological systems such as proteins [34]. Thus, compound **9** could be an interesting potential model structure for the exploration of chalcogen–π ChB.

Further, we explored the influence of charged (cationic) substituents by the nitrile group on the cyclization with 2-pyridylselenylchloride. *p*-Cyano-*N*-methylpyridinium methanesulphonate and *o*-cyanodiazonium tetrafluoroborate readily reacted with 2-pyridylselenylchloride, which resulted in the formation of adducts **7** and **10** in high yields. Interestingly, the presence of the diazonium group did not affect the cyclization, demonstrating a remarkable functional group tolerance of the coupling with nitriles. 

**Figure 7:**
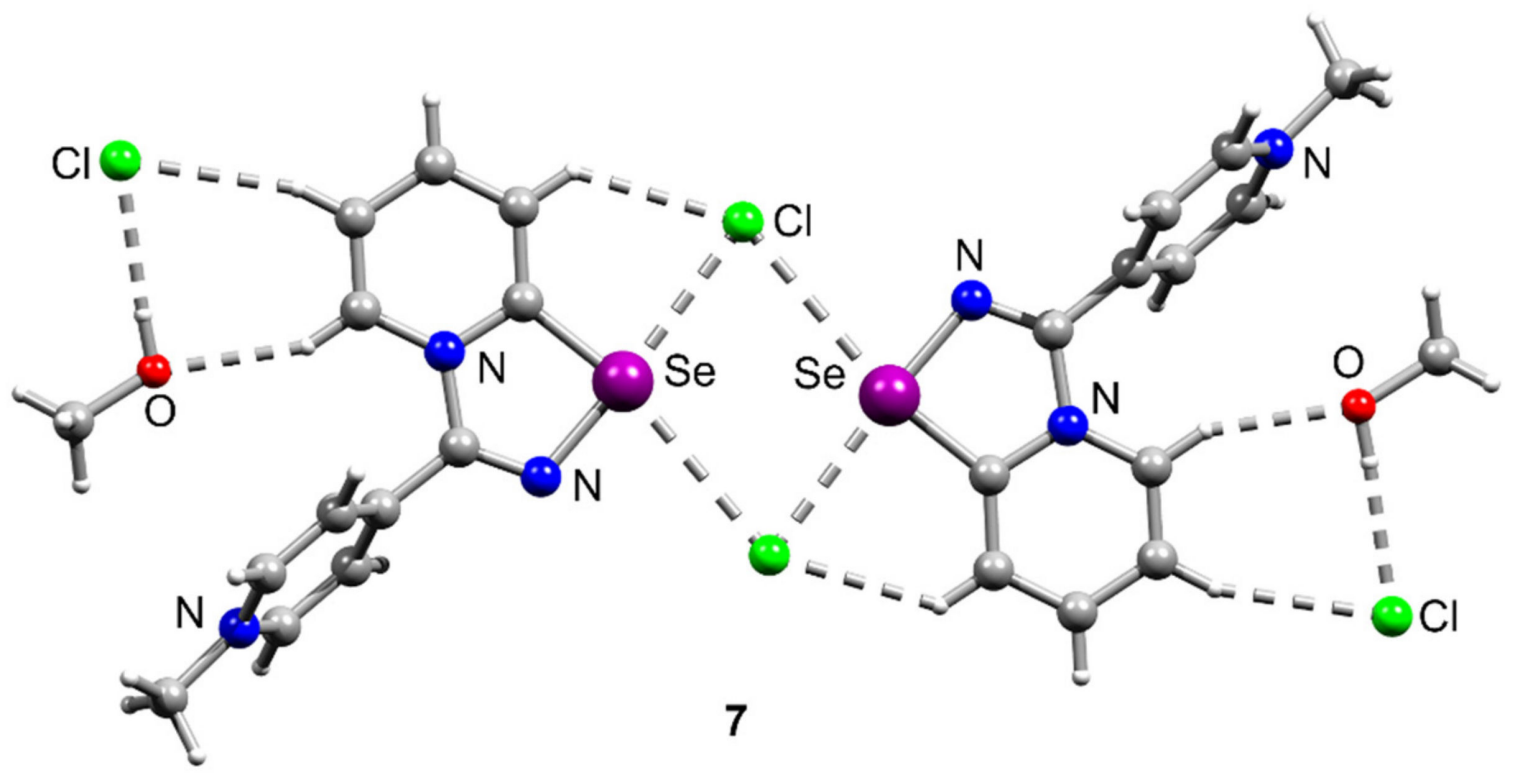
Ball-and-stick representation of the crystal structure of **7**, demonstrating attractive Se∙∙∙Cl, H∙∙∙Cl, and H∙∙∙O interactions. Grey and light-grey spheres represent carbon and hydrogen, respectively.

The structural analysis revealed that dicationic 1,2,4-selenadiazoles in **7** formed [Se–Cl]_2_ squares (Figure 7), which we observed earlier [22]. It should be noted that the second chloride did not participate in ChB (Figure 7). 

**Figure 8:**
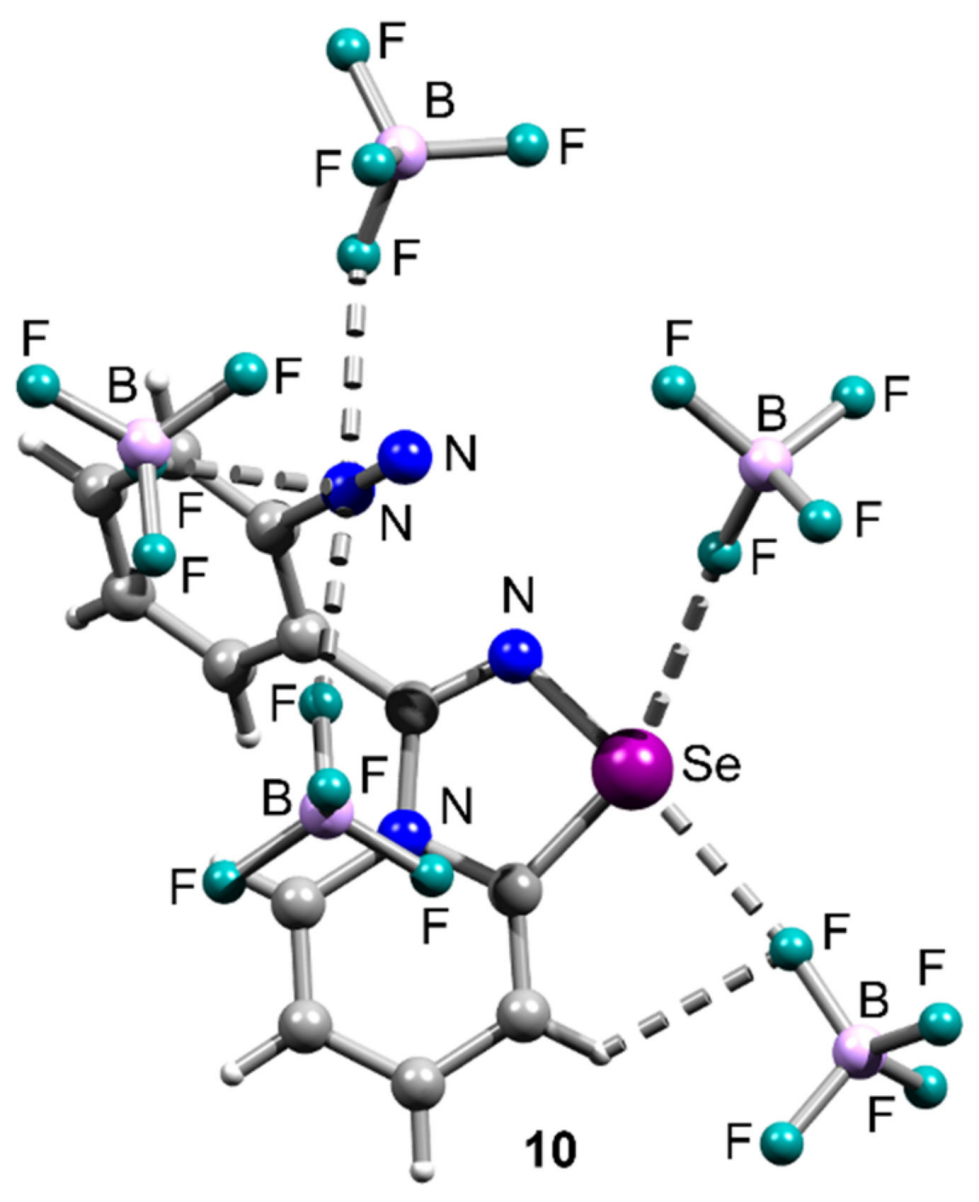
Ball-and-stick representation of the crystal structure of **10**, demonstrating attractive Se∙∙∙F, H∙∙∙F, and N∙∙∙F interactions. Grey and light-grey spheres represent carbon and hydrogen, respectively.

The adduct **10** featuring diazonium moiety and two BF_4_ anions did not form supramolecular dimers or polymers in the solid state (Figure 8). The Se center was involved in two Se∙∙∙F ChBs, while the N_2_^+^ group—in three N∙∙∙F pnictogen bonding interactions (Figure 8).

**Figure 9:**
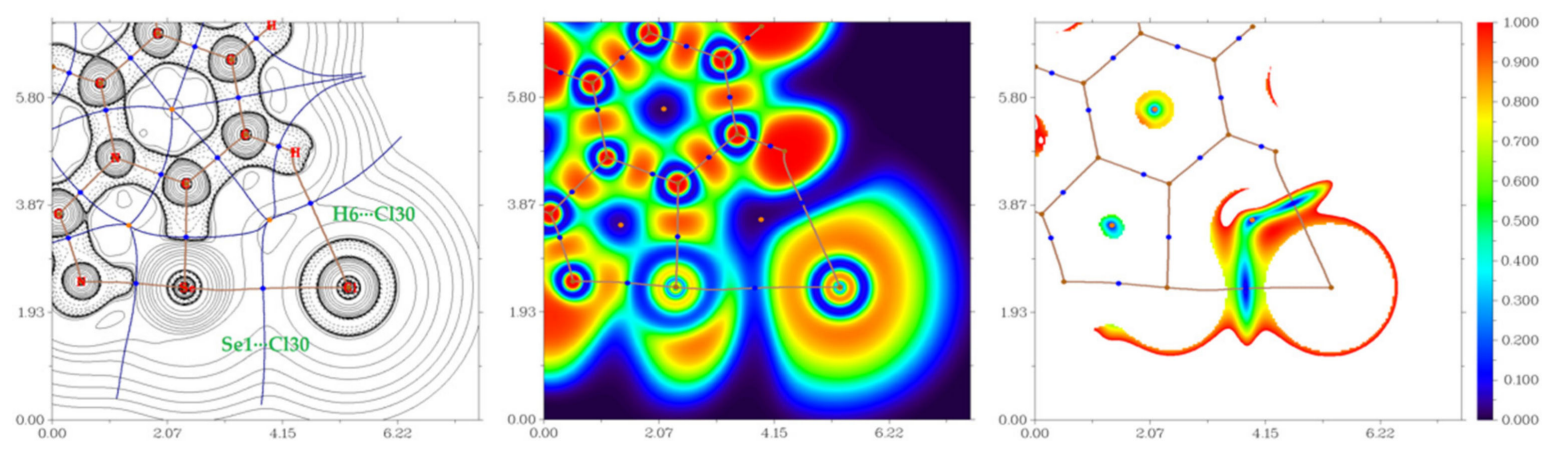
Contour line diagram of the Laplacian of electron density distribution ∇^2^*ρ*(**r**), bond paths, and selected zero-flux surfaces (left panel), and visualization of electron localization function (ELF, center panel) and reduced density gradient (RDG, right panel) analyses for non-covalent interactions H6···Cl30 and Se1···Cl30 in the model supramolecular associate **4**. Bond critical points (3, −1) are shown in blue, nuclear critical points (3, −3) in pale brown, ring critical points in orange. Bond paths are shown as pale brown lines, length units are in Å, and the color scale for the ELF and RDG maps is presented in a.u. The numeration of atoms corresponds to their ordering in the attached xyz-files for model supramolecular associates (Supplementary Materials).

Inspection of the crystallographic data revealed the presence of various non-trivial non-covalent interactions in the crystal structures of **3**–**10**. To understand the nature and quantify strength of these non-covalent interactions, DFT calculations followed by a topological analysis of the electron-density distribution within the QTAIM approach [35] were carried out at the ωB97X-D3/Sapporo-DZP-2012 level of theory for model supramolecular associates (see Computational Details in the Materials and Methods, and the attached xyz-files in the Supplementary Materials). The results of the QTAIM analysis are summarized in Table S2 (the Poincaré–Hopf relationship was satisfied in all cases). The contour line diagrams of the Laplacian of electron density distribution ∇^2^*ρ*(r), bond paths, selected zero-flux surfaces, visualization of electron localization function (ELF), and reduced density gradient (RDG) analyses for some of these non-covalent interactions are shown in Figure 9 and Figures S1–S6.

The QTAIM analysis of model supramolecular associates **3**–**10** demonstrated the presence of bond critical points (3, –1) for the non-covalent interactions listed in Table S2 and shown in Figure 9 and Figures S1–S6. In all cases, with the exception of Se25···Cl27 and Se25···Cl26 contacts in **6**, the low magnitude of the electron density (0.007–0.028 a.u.), positive values of the Laplacian of electron density (0.020–0.066 a.u.), zero or very close to zero energy density (0.000–0.002 a.u.) in these bond critical points (3, –1), and estimated strength for appropriate short contacts (0.9–5.0 kcal/mol) are typical for weak hydrogen bonds [3,4,36] and non-covalent interactions involving halogen [8,9,10,11,12,32,37,38] and chalcogen [22,23,39,40,41,42] atoms in similar chemical systems. The strongest non-covalent interactions in the studied model supramolecular associates **3**–**10** were chalcogen bonds: Se1···Cl24 in **3** (4.7 kcal/mol), Se1···Cl30 in **4** (4.4 kcal/mol), Se1···Cl38 in **5** (5.0 kcal/mol), Se1···Cl24 in **6** (3.5 kcal/mol), Se29···Cl56 in **7** (5.0 kcal/mol), Se1···Br15 in **8** (3.5 kcal/mol), Se2···C31 in **9** (1.3 kcal/mol), and Se1···F29 (4.7 kcal/mol) in **10**. In cases of Se25···Cl27 and Se25···Cl26 contacts in **6**, the significant magnitude of the electron density (0.064 and 0.069 a.u.), clear negative energy density (−0.015 and −0.018), and relatively high estimated strength (14.7 and 16.6 kcal/mol) allow us to consider these closed-shell interactions more like coordination bonds rather than non-covalent interactions [43,44]. Note that the energies of non-covalent interactions can also be potentially estimated on the basis of the experimental electron-density distribution functions [45,46]. The balance between the Lagrangian kinetic energy G(r) and potential energy density V(r) at the bond critical points (3, −1) reveals the nature of these interactions: if the ratio −G(r)/V(r) > 1 is satisfied, then the nature of appropriate interaction is purely non-covalent, whereas in the case of –G(**r**)/V(**r**) < 1, some covalent component takes place [47]. On the basis of this criterion, one can state that a covalent contribution in all interactions listed in Table S2, except Se25···Cl27 and Se25···Cl26 contacts in **6**, was absent, whereas Se25···Cl27 and Se25···Cl26 contacts in **6** had significant degrees of covalency. The Laplacian of electron density is typically decomposed into the sum of contributions along the three principal axes of maximal variation, giving the three eigenvalues of the Hessian matrix (λ_1_, λ_2_ and λ_3_), and the sign of λ_2_ can be utilized to distinguish bonding (attractive, λ_2_ < 0) weak interactions from nonbonding ones (repulsive, λ_2_ > 0) [48,49]. Thus, all contacts listed in Table S2 are attractive.

## 3. Materials and Methods

### 3.1. General Remarks 

All manipulations were carried out in air unless specified otherwise. All reagents used in this study were obtained from commercial sources (Aldrich, TCI-Europe, Strem, ABCR). Commercially available solvents were purified by conventional methods and distilled immediately prior to use. NMR spectra were recorded on a Bruker Avance III (Karlsruhe, Germany). Chemical shifts (*δ*) are given in ppm, coupling constants (*J*) are given in Hz. C, H, S, and N elemental analyses were carried out on a Euro EA 3028HT CHNS/O analyzer (Pavia, Italy). 2-Pyridylselenyl chloride, 2-pyridylselenyl bromide, and di-(2-pyridyl)-diselenide were synthesized as reported earlier [50,51].

### 3.2. X-ray Crystal Structure Determination 

The single-crystal X-ray diffraction data for **3**–**10** were collected on three-circle Bruker D8 Venture or Bruker Smart Apex II diffractometers (Centre of Joint Equipment of Kurnakov Institute of General and Inorganic Chemistry, Russian Academy of Sciences) using *φ* and *ω* scan modes. The data were indexed and integrated using the SAINT program [52]. Absorption corrections based on measurements of equivalent reflections (SADABS) were applied [53]. The structures were determined by direct methods and refined by a full-matrix least squares technique on F2 with anisotropic displacement parameters for non-hydrogen atoms. The hydrogen atoms in all compounds were placed in calculated positions and refined within riding models with fixed isotropic displacement parameters (Uiso(H) = 1.5Ueq(C) for the CH3-groups and 1.2Ueq(C) for the other groups). All calculations were carried out using the SHELXTL program [54] and OLEX2 program package [55]. For details, see Table S1 (Supplementary Materials).

Crystallographic data for all investigated compounds have been deposited with the Cambridge Crystallographic Data Center, CCDC 2129995-2130002. Copies of this information may be obtained free of charge from the Director, CCDC, 12 Union Road, Cambridge, CHB2 1EZ, UK (Fax: +44 1223 336033; e-mail: deposit@ccdc.cam.ac.uk; or www.ccdc.cam.ac.uk).

### 3.3. Computational Details

The DFT calculations based on the experimental X-ray geometries of **3**–**10** were carried out at the ωB97X-D3/Sapporo-DZP-2012 level of theory [56,57,58,59] with the help of the ORCA 4.2.1 program package [60]. The RIJCOSX approximation [61] was utilized. The topological analysis of the electron density distribution, with the help of the QTAIM approach [35], was performed using the Multiwfn program (version 3.7) [62]. The Cartesian atomic coordinates for model supramolecular associates are presented in the attached xyz-files in the Supplementary Materials.

### 3.4. Synthesis of Compounds ***3***–***5*** and ***7***–***10***

**3**. 2-Pyridylselenyl chloride (182 µmol, 35 mg) and butyronitrile (690 µmol, 60 µL) were stirred in Et_2_O (4 mL) at room temperature for 12 h. A colorless precipitate gradually formed and was filtered, washed with Et_2_O (3 × 3 mL), and dried under vacuum. Yield: 37 mg (78%). Anal. Calcd. for C_9_H_11_ClN_2_Se: C, 41.32; H, 4.24; N, 10.71. Found: C, 41.41; H, 4.47; N, 10.64. ^1^H NMR (600 MHz, D_2_O) *δ* 9.37 (1H, dt, *J* = 6.9, 0.9 Hz, H5), 8.80 (1H, dt, *J* = 8.7, 1.0 Hz, H8), 8.39 (1H, ddd, *J* = 8.5, 7.2, 1.1 Hz, H7), 8.01 (1H, td, *J* = 7.0, 1.2 Hz, H6), 3.31 (2H, t, *J* = 7.4 Hz, CH_2_), 2.02 (2H, h, *J* = 7.4 Hz, CH_2_), 1.10 (3H, t, *J* = 7.4 Hz, CH_3_). ^13^C{^1^H} NMR *δ* 167.8 (C3), 159.1 (C9), 139.5 (C5), 136.0 (C8), 125.9 (C7), 123.0 (C6), 32.8 (CH_2_), 18.4 (CH_2_), 12.8 (CH_3_). Crystals suitable for X-ray analysis were obtained by the slow evaporation of the saturated CH_2_Cl_2_ solution.

**4**. 2-Pyridylselenyl chloride (208 µmol, 40 mg) was stirred with hexanenitrile (100 µL) in Et_2_O (mL) at room temperature for 12 h. A colorless precipitate gradually formed and was filtered, washed with Et_2_O (3 × 3 mL), and dried under vacuum. Yield: 63 mg (94%). Anal. Calcd. for C_11_H_15_ClN_2_Se: C, 45.61; H, 5.22; N, 9.67. Found: C, 45.58; H, 5.34; N, 9.64. ^1^H NMR (600 MHz, D_2_O) *δ* 9.35 (1H, d, *J* = 6.8 Hz, H5), 8.79 (1H, d, *J* = 8.7 Hz, H8), 8.38 (1H, t, *J* = 7.9 Hz, H7), 8.00 (1H, t, *J* = 7.0 Hz, H6), 3.32 (2H, t, *J* = 7.5 Hz, CH_2_), 1.98 (2H, p, *J* = 7.5 Hz, CH_2_), 1.47 (2H, p, *J* = 7.4 Hz, CH_2_), 1.38 (2H, dq, *J* = 14.8, 7.4 Hz, CH_2_), 0.88 (3H, t, *J* = 7.4 Hz, CH_3_). ^13^C{^1^H} NMR *δ* 167.7 (C3), 159.2 (C9), 139.4 (C5), 135.9 (C8), 125.8 (C7), 122.9 (C6), 30.8 (CH_2_), 30.3 (CH_2_), 24.3 (CH_2_), 21.6 (CH_2_), 13.1 (CH_3_). Crystals suitable for X-ray analysis were obtained by the slow evaporation of the saturated MeOH solution.

**5**. 2-Pyridylselenyl chloride (130 µmol, 25 mg) was stirred with 2,2-diphenylacetonitrile (264 µmol, 51 mg) in Et_2_O (5 mL) at room temperature for 3 h. A colorless precipitate gradually formed and was filtered, washed with CH_2_Cl_2_ (3 mL), then Et_2_O (3×3 mL), and dried under vacuum. Yield: 42 mg (84%). Anal. Calcd. for C_19_H_15_ClN_2_Se: C, 59.16; H, 3.92; N, 7.26. Found: C, 59.38; H, 4.12; N, 7.21. ^1^H NMR (600 MHz, D_2_O) *δ* 9.17 (1H, d, J = 6.9 Hz, H5), 8.83 (1H, d, J = 8.6 Hz, H8), 8.69 (5H, t, J = 6.4 Hz, 5H from Ph), 8.34 (1H, t, J = 7.9 Hz, H7), 7.76–7.72 (1H, m, H6), 7.45–7.36 (5H, m, 5H from Ph), 6.40 (1H, s, CH). ^13^C{^1^H} *δ* 168.6 (C3), 158.6 (C9),140.8 (C11 and C11′), 139.7 (C5), 136.1 (C8), 129.0 (C13 and C15 and C13′ and C15′), 128.5 (C12 and C16 and C12′ and C16′), 127.8 (C14 and C14′), 126.2 (C7), 123.1 (C6), 52.9 (C10). Crystals suitable for X-ray analysis were obtained by the slow evaporation of the saturated CH_2_Cl_2_ solution.

**7**. 2-Pyridylselenyl chloride (104 µmol, 20 mg), 4-cyano-N-methylpyridinium methanesulfonate (104 µmol, 22 mg), and Bu_4_NCl (360 µmol, 100 mg) were stirred in MeOH/CH_2_Cl_2_ (2 mL/2 mL) at room temperature for 12 h. A colorless precipitate gradually formed and was filtered, washed with CH_2_Cl_2_ (3 × 3 mL), then Et_2_O (3 × 3 mL), and dried under vacuum. Yield: 28 mg (86%). Anal. Calcd. for C_12_H_11_Cl_2_N_3_Se: C, 41.52; H, 3.19; N, 12.11. Found: C, 41.74; H, 3.38; N, 12.09. ^1^H NMR (600 MHz, D_2_O) *δ* 9.30 (1H, d, *J* = 6.8 Hz, H5), 9.24 (2H, d, *J* = 6.5 Hz, H11 and H14), 8.98 (1H, d, *J* = 8.7 Hz, H8), 8.56 (2H, d, *J* = 6.2 Hz, H12 and H13), 8.51 (1H, t, *J* = 7.9 Hz, H7), 8.06 (1H, t, *J* = 7.0 Hz, H6), 4.59 (3H, s, CH_3_). ^13^C{^1^H} *δ* 169.2 (C3), 150.9 (C9), 147.2 (C5), 143.1 (C10), 140.4 (C8), 136.3 (C11 and C14), 128.7 (C7), 126.5 (C12 and C13), 123.8 (C6), 38.4 (N–CH_3_). Crystals suitable for X-ray analysis were obtained by the slow evaporation of the saturated MeOH solution.

**8**. 2-Pyridylselenyl bromide (245 µmol, 58 mg) was stirred with cyanogen bromide (1.22 mmol, 45 mg) in Et_2_O (4 mL) at room temperature for 3 h. A light-yellow precipitate gradually formed and was filtered, washed with Et_2_O (3 × 3 mL), then hexane (3 × 3 mL), and dried under vacuum. Yield: 63 mg (75%). Anal. Calcd. For C_6_H_4_Br_2_N_2_Se: C, 21.02; H, 1.18; N, 8.17. Found: C, 21.15; N, 8.22. ^1^H NMR (600 MHz, D_2_O) *δ* 9.61 (1H, d, *J* = 6.9 Hz, H5), 8.88 (1H, d, *J* = 8.7 Hz, H8), 8.51–8.44 (1H, m, H7), 8.11 (1H, td, J = 7.0, 1.0 Hz, H6). ^13^C{^1^H} NMR *δ* 168.4 (C3), 146.4 (C9), 140.6 (C5), 137.7 (C8), 126.3 (C7), 123.7 (C6). Crystals suitable for X-ray analysis were obtained by the slow evaporation of the saturated CH_2_Cl_2_ solution.

**9.** NaBPh4 (58 µmol, 20 mg) in MeOH (1 mL) was added to the solution of 8 (58 µmol, 20 mg) in MeOH (2 mL), and the mixture was stirred at room temperature for 30 min. Colorless precipitate gradually formed and was filtered, washed with Et2O (3 × 3 mL), and dried under vacuum. Anal. Calcd. for C30H24BBrN2Se: C, 61.89; H, 4.16; N, 4.81. Found: C, 62.05; H, 4.31; N, 4.73 1H and 13C NMR were not recorded due to the insolubility of 9 in D2O. Crystals suitable for X-ray analysis were obtained by the slow evaporation of the saturated MeOH solution.

**10**. 2-Pyridylselenyl chloride (208 µmol, 40 mg) was stirred with 2-cyanobenzenediazonium tetrafluoroborate (228 µmol, 50 mg) and KBF_4_ (397 µmol, 50 mg) in MeOH (4 mL) at room temperature for 3 h. A pale-orange precipitate gradually formed and was filtered, washed with Et_2_O (3 × 3 mL), and dried under vacuum. Yield: 50 mg (52%). Anal. Calcd. for C_12_H_8_B_2_F_8_N_4_Se: C, 31.28; H, 1.75; N, 12.16. Found: C, 31.34; H, 2.16; N, 11.98 ^1^H NMR (600 MHz, DMSO-*d*_6_) δ 9.48 (1H, d, *J* = 6.8 Hz, H5), 9.11 (1H, d, *J* = 8.7 Hz, H14), 9.06 (1H, dd, *J* = 8.4, 0.9 Hz, H11), 8.61 (1H, td, *J* = 7.8, 1.0 Hz, H12), 8.58–8.53 (1H, m, H13), 8.53–8.48 (1H, m, H8), 8.39–8.34 (1H, m, H7), 8.05 (1H, td, *J* = 7.0, 1.0 Hz, H6). ^13^C{^1^H} NMR *δ* 169.0 (C3), 148.2 (C9), 141.6 (C5), 140.4 (C8), 137.8 (C15), 136.7 (C11), 134.0 (C13), 132.7 (C12), 129.1 (C14), 126.6 (C7), 123.1 (C6), 116.3 (C10). Crystals suitable for X-ray analysis were obtained by the slow evaporation of the saturated MeOH solution.

## 4. Conclusions

In summary, eight novel cationic 1,2,4-selenadiazoles were structurally characterized. Solid state structures exhibited multiple ChB interactions, which were studied by DFT calculations and topological analysis of the electron-density distribution within the framework of Bader’s theory (QTAIM method). Formation of [Se–N]_2_ squares, which we observed earlier, did not occur for selenadiazoles studied within this work. Although substituent-dependent self-assembly in the solid state was observed, a priori prediction of the packing preference is still challenging due to the presence of multiple competing weak interactions. Overall, we demonstrated several new types of structural organization of novel cationic 1,2,4-selenadiazole building blocks in the solid state, which involved attractive ChB interactions. Further studies into the chemistry of cationic 1,2,4-selenadiazoles and their applications from our laboratory are underway, and will be reported in due course.

## Supplementary Materials

The following supporting information can be downloaded: Table S1 with crystal data and structure refinements for **3**–**10**; Table S2 with details of QTAIM analysis [63,64]; Figures S1–S6 with contour line diagrams of the Laplacian of electron density distribution ∇^2^*ρ*(**r**), bond paths, selected zero-flux surfaces, visualization of electron localization function (ELF), and reduced density gradient (RDG) analyses for some of non-covalent interactions in **5**, **6**, **8**, **9**, and **10**; xyz-files with Cartesian atomic coordinates for model supramolecular associates.

## Sample Availability

Samples of compounds **3**–**10** are available from the authors.
