# Supplementary material for: Exploring Supramolecular Assembly Space of Cationic 1,2,4-Selenodiazoles: Effect of the Substituent at the Carbon Atom and Anions
Source: Molecules. 2022 Feb 2;27(3):1029. doi: 10.3390/molecules27031029 (PMC8839610; doi:10.3390/molecules27031029)
Supplement: Supplementary file 1 [file molecules-27-01029-s001.zip › molecules-1550745-supplementary.pdf]

Supplementary Materials

Exploring Supramolecular Assembly Space of Cationic 1,2,4-Selenadiazoles: Effect of the Substituent at the Carbon Atom and Anions

Mariya V. Grudova,[1] Alexey S. Kubasov,[2] Victor N. Khrustalev,[1,3] Alexander S. Novikov,[4] Andreii S. Kritchenkov,[1] Valentine G. Nenajdenko,[5] Alexander V. Borisov,[6] and Alexander G. Tskhovrebov[1,7]*

1 Peoples' Friendship University of Russia, 6 Miklukho-Maklaya Street, Moscow, 117198, Russian Federation

2 Kurnakov Institute of General and Inorganic Chemistry, Russian Academy of Sciences, Leninsky Prosp. 31, Moscow, Russian Federation

3 N.D. Zelinsky Institute of Organic Chemistry, Russian Academy of Sciences, 47 Leninsky Prosp., Moscow, Russian Federation

4 Saint Petersburg State University, Universitetskaya Nab. 7/9, Saint Petersburg, Russian Federation

5 Lomonosov Moscow State University, Leninskie Gory 1/3, Moscow, Russian Federation

6 R.E. Alekseev Nizhny Novgorod State Technical University, Minin St., 24, Nizhny Novgorod, Russian Federation.

7 N.N. Semenov Federal Research Center for Chemical Physics, Russian Academy of Sciences, Ul. Kosygina 4, Moscow, Russian Federation

Table S1. Crystal data and structure refinements for **3-10**.

Compound	**3**	**4**	**5**
Empirical formula	$C_9H_{13}ClN_2OSe$	$C_{11}H_{17}ClN_2OSe$	$C_{20}H_{17}Cl_3N_2Se$
Formula weight	279.62	307.67	470.66
Temperature/K	100.0	100.00	150
Crystal system	monoclinic	monoclinic	monoclinic
Space group	$P2_1/c$	$P2_1/c$	$P2_1/c$
a/Å	6.6225(19)	6.729(5)	8.673(2)
b/Å	34.267(13)	40.39(3)	17.696(5)
c/Å	5.0296(12)	4.971(3)	13.107(5)
β/°	104.149(9)	104.006(14)	105.67(2)
Volume/Å^3	1106.8(6)	1310.6(15)	1936.8(10)
Z	4	4	4
ρ_{calc}g/cm^3	1.678	1.559	1.614
μ/mm^{-1}	3.604	3.051	2.359
F(000)	560.0	624.0	944.0
Radiation	MoKα (λ = 0.71073)	MoKα (λ = 0.71073)	MoKα (λ = 0.71073)
2Θ range for data collection/°	6.344 to 60.35	4.034 to 60.1	4.604 to 60.166
Reflections collected	7355	7461	17180
Independent reflections	3219 [R_{int} = 0.0408, R_{sigma} = 0.0556]	3743 [R_{int} = 0.0328, R_{sigma} = 0.0583]	5238 [R_{int} = 0.0372, R_{sigma} = 0.0425]
Data/restraints/parameters	3219/0/131	3743/0/143	5238/0/235
Goodness-of-fit on F^2	1.036	1.246	1.033
Final R indexes [I>=2σ (I)]	R_1 = 0.0360, wR_2 = 0.0860	R_1 = 0.0652, wR_2 = 0.1119	R_1 = 0.0316, wR_2 = 0.0600
Final R indexes [all data]	R_1 = 0.0440, wR_2 = 0.0894	R_1 = 0.0835, wR_2 = 0.1170	R_1 = 0.0462, wR_2 = 0.0662
Largest diff. peak/hole / e Å^{-3}	0.95/-0.67	0.81/-1.45	0.35/-0.46

Compound	**6**	**7**	**8**
Empirical formula	$C_{14}H_{16}Cl_3N_3Se_2$	$C_{13}H_{15}Cl_2N_3OSe$	$C_6H_4Br_2N_2Se$
Formula weight	490.57	379.14	342.89
Temperature/K	150	150.00	150.00
Crystal system	triclinic	triclinic	tetragonal
Space group	P-1	P-1	$I4_1cd$
a/Å	7.370(4)	7.1031(18)	15.8426(4)
b/Å	9.175(3)	10.061(3)	15.8426(4)
c/Å	13.763(5)	11.734(3)	13.3489(4)
α/°	80.229(11)	96.704(11)	90
β/°	89.20(2)	106.120(10)	90
γ/°	83.08(2)	101.145(11)	90
Volume/Å^3	910.5(6)	777.4(4)	3350.4(2)

Z	2	2	16
ρ_{calc}g/cm^3	1.789	1.620	2.719
μ/mm^{-1}	4.499	2.757	13.954
F(000)	480.0	380.0	2528.0
Radiation	MoKα (λ = 0.71073)	MoKα (λ = 0.71073)	MoKα (λ = 0.71073)
2Θ range for data collection/°	3.002 to 55.674	3.674 to 67.198	5.142 to 67.192
Reflections collected	8050	9460	10217
Independent reflections	4213 [R_{int} = 0.0397, R_{sigma} = 0.0610]	5301 [R_{int} = 0.0182, R_{sigma} = 0.0331]	2969 [R_{int} = 0.0332, R_{sigma} = 0.0371]
Data/restraints/parameters	4213/0/200	5301/0/184	2969/1/100
Goodness-of-fit on F^2	1.000	1.023	1.036
Final R indexes [I>=2σ (I)]	R_1 = 0.0351, wR_2 = 0.0838	R_1 = 0.0275, wR_2 = 0.0572	R_1 = 0.0425, wR_2 = 0.1065
Final R indexes [all data]	R_1 = 0.0439, wR_2 = 0.0884	R_1 = 0.0355, wR_2 = 0.0599	R_1 = 0.0547, wR_2 = 0.1128
Largest diff. peak/hole / e Å^{-3}	0.46/-0.72	0.45/-0.34	1.86/-1.10
			0.474(13)

Compound	**9**	**10**
Empirical formula	$C_{30}H_{24}BBrN_2Se$	$C_{12}H_8B_2F_8N_4Se$
Formula weight	582.19	460.80
Temperature/K	150.00	100
Crystal system	tetragonal	monoclinic
Space group	$P4_12_12$	$P2_1/c$
a/Å	10.045	14.880(17)
b/Å	10.045	10.729(9)
c/Å	50.090	20.07(2)
β/°	90	98.16(4)
Volume/Å^3	5054.0	3172(6)
Z	8	8
ρ_{calc}g/cm^3	1.530	1.930
μ/mm^{-1}	3.089	2.460
F(000)	2336.0	1792.0
Radiation	MoKα (λ = 0.71073)	MoKα (λ = 0.71073)
2Θ range for data collection/°	4.136 to 54.696	4.1 to 60.334
Reflections collected	18377	30019
Independent reflections	5561 [R_{int} = 0.0404, R_{sigma} = 0.0562]	8445 [R_{int} = 0.0647, R_{sigma} = 0.0690]
Data/restraints/parameters	5561/0/316	8445/0/487
Goodness-of-fit on F^2	1.037	1.016
Final R indexes [I>=2σ (I)]	R_1 = 0.0370, wR_2 = 0.0711	R_1 = 0.0501, wR_2 = 0.1060
Final R indexes [all data]	R_1 = 0.0507, wR_2 = 0.0753	R_1 = 0.0852, wR_2 = 0.1226

Largest diff. peak/hole / e Å^{-3}	0.43/-0.61	0.79/-1.09
Flack parameter	0.057(6)	

Table S2. Values of the density of all electrons – ρ(**r**), Laplacian of electron density – ∇^2ρ(**r**) and appropriate λ_2 eigenvalues, energy density – H_b, potential energy density – V(**r**), and Lagrangian kinetic energy – G(**r**) (a.u.) at the bond critical points (3, –1), corresponding to various nontrivial non-covalent interactions in the model supramolecular associates **3**–**10**, and estimated strength for these contacts E_{int} (kcal/mol).

Contact*	ρ(**r**)	∇^2ρ(**r**)	λ_2	H_b	V(**r**)	G(**r**)	E_{int}**
**3**							
H10···Cl24 2.646 Å	0.012	0.041	-0.012	0.001	-0.008	0.009	2.5
Se1···Cl24 2.930 Å	0.027	0.057	-0.027	0.000	-0.015	0.015	4.7
**4**							
H6···Cl30 2.616 Å	0.013	0.043	-0.013	0.001	-0.008	0.009	2.5
Se1···Cl30 2.957 Å	0.026	0.055	-0.026	0.000	-0.014	0.014	4.4
**5**							
H4···Cl38 2.601 Å	0.014	0.045	-0.014	0.001	-0.009	0.010	2.8
Se1···Cl38 2.902 Å	0.028	0.060	-0.028	0.000	-0.016	0.016	5.0
H43···N5 2.698 Å	0.008	0.031	-0.008	0.002	-0.005	0.006	1.6
Se1···Cl39 3.423 Å	0.009	0.031	-0.009	0.001	-0.005	0.006	1.6
**6**							
H6···Cl24 2.706 Å	0.011	0.035	-0.011	0.001	-0.007	0.008	2.2
Se1···Cl24 3.063 Å	0.021	0.049	-0.021	0.001	-0.011	0.012	3.5
Se1···Cl27 3.203 Å	0.014	0.045	-0.014	0.001	-0.008	0.010	2.5
Se25···N3 3.417 Å	0.007	0.020	-0.007	0.001	-0.004	0.004	1.3
Se25···Cl27 2.472 Å	0.064	0.068	-0.064	-0.015	-0.047	0.032	14.7
Se25···Cl26 2.431 Å	0.069	0.066	-0.069	-0.018	-0.053	0.035	16.6
**7**							

H40···Cl56 2.616 Å	0.013	0.043	-0.013	0.001	-0.008	0.009	2.5
Se29···Cl56 2.938 Å	0.026	0.062	-0.026	0.000	-0.016	0.016	5.0
Se29···Cl28 3.337 Å	0.012	0.034	-0.012	0.001	-0.006	0.007	1.9
			**8**				
Br31···Br15 3.678 Å	0.008	0.021	-0.008	0.001	-0.003	0.004	0.9
Se1···Br15 3.149 Å	0.021	0.046	-0.021	0.000	-0.011	0.011	3.5
Se16···Br15 3.434 Å	0.012	0.031	-0.012	0.001	-0.006	0.007	1.9
H13···Br15 2.731 Å	0.013	0.036	-0.013	0.001	-0.008	0.008	2.5
H13···Br31 2.823 Å	0.008	0.030	-0.008	0.001	-0.005	0.006	1.6
			**9**				
Se2···C31 3.257 Å	0.010	0.028	-0.010	0.001	-0.004	0.006	1.3
Se61···C42 3.300 Å	0.010	0.025	-0.010	0.001	-0.004	0.005	1.3
			**10**				
H8···F29 2.472 Å	0.009	0.038	-0.009	0.001	-0.007	0.008	2.2
Se1···F29 2.723 Å	0.018	0.066	-0.018	0.001	-0.015	0.016	4.7
Se1···F36 3.061 Å	0.009	0.036	-0.009	0.001	-0.007	0.008	2.2
F43···N4 2.755 Å	0.010	0.046	-0.010	0.002	-0.008	0.010	2.5
F49···N4 2.769 Å	0.010	0.045	-0.010	0.002	-0.008	0.009	2.5
F32···N4 2.839 Å	0.009	0.039	-0.009	0.002	-0.006	0.008	1.9

* The Bondi's van der Waals radii for H, C, N, F, Cl, Br, and Se atoms are 1.20, 1.70, 1.55, 1.47, 1.75, 1.83, and 1.90 Å, respectively.[Bondi, A. Van der Waals volumes and radii of metals in covalent compounds. *J. Phys. Chem.* **1966**, *70*, 3006–3007, doi:10.1021/j100881a503.] The numeration of atoms corresponds to their ordering in the attached xyz-files for model supramolecular associates (Supplementary materials).

** $E_{int} \approx -V(\mathbf{r})/2$[Espinosa, E.; Molins, E.; Lecomte, C. Hydrogen bond strengths revealed by topological analyses of experimentally observed electron densities. *Chem. Phys. Lett.* **1998**, *285*, 170–173, doi:10.1016/S0009-2614(98)00036-0.]

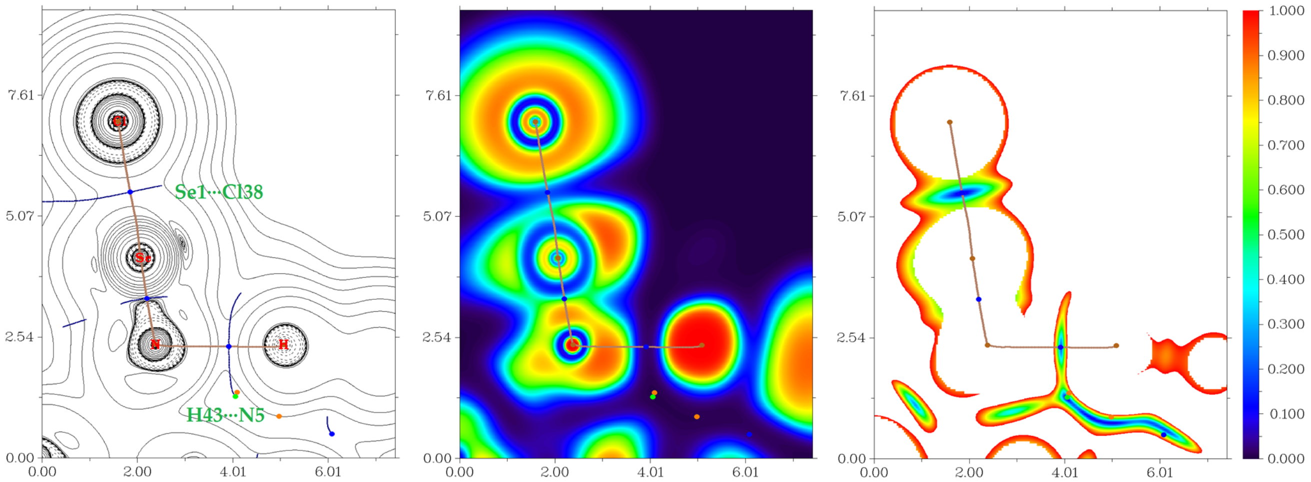

Figure S1. Contour line diagram of the Laplacian of electron density distribution $\nabla^2\rho(\mathbf{r})$, bond paths, and selected zero-flux surfaces (left panel), visualization of electron localization function (ELF, center panel) and reduced density gradient (RDG, right panel) analyses for non-covalent interactions H43···N5 and Se1···Cl38 in the model supramolecular associate **5**. Bond critical points (3, –1) are shown in blue, nuclear critical points (3, –3) – in pale brown, ring critical points – in orange, cage critical points (3, +3) – in light green, bond paths are shown as pale brown lines, length units – Å, and the color scale for the ELF and RDG maps is presented in a.u. The numeration of atoms corresponds to their ordering in the attached xyz-files for model supramolecular associates (Supplementary materials).

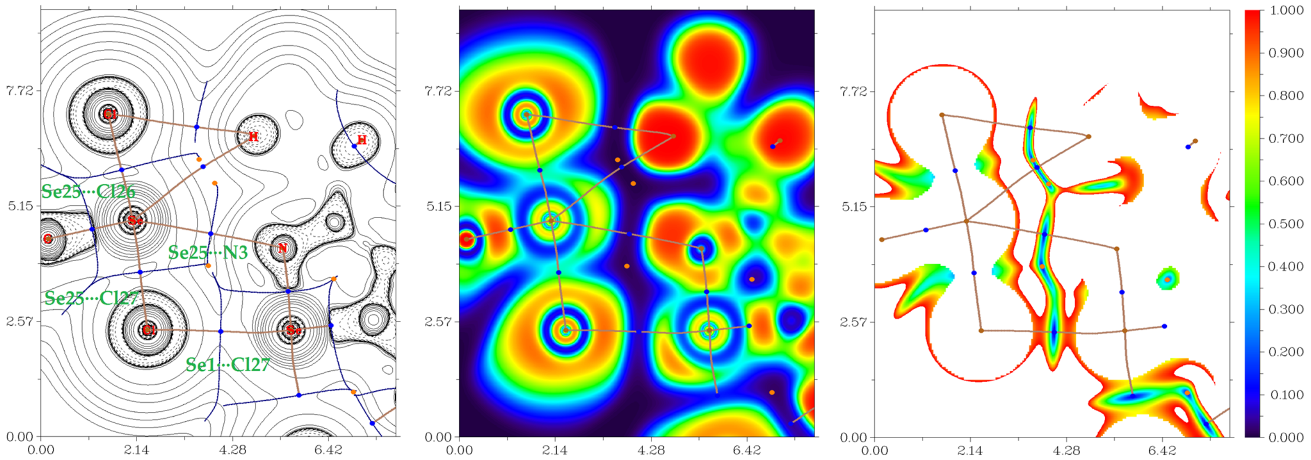

Figure S2. Contour line diagram of the Laplacian of electron density distribution $\nabla^2\rho(\mathbf{r})$, bond paths, and selected zero-flux surfaces (left panel), visualization of electron localization function (ELF, center panel) and reduced density gradient (RDG, right panel) analyses for non-covalent interactions Se1···Cl27, Se25···N3, Se25···Cl27, and Se25···Cl26 in the model supramolecular associate **6**. Bond critical points (3, –1) are shown in blue, nuclear critical points (3, –3) – in pale brown, ring critical points – in orange, bond paths are shown as pale brown lines, length units – Å, and the color scale for the ELF and RDG maps is presented in a.u. The numeration of atoms corresponds to their ordering in the attached xyz-files for model supramolecular associates (Supplementary materials).

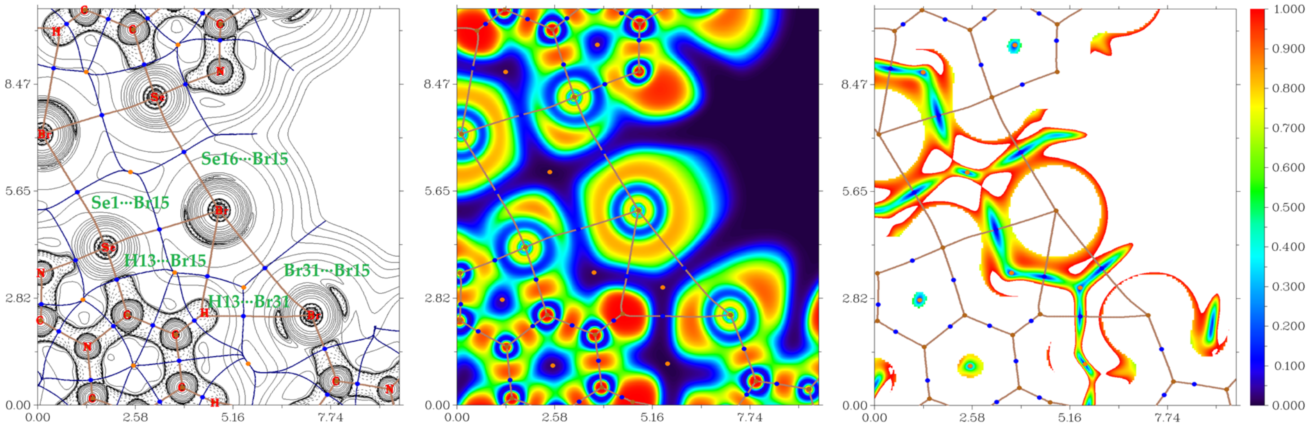

Figure S3. Contour line diagram of the Laplacian of electron density distribution ∇²ρ(**r**), bond paths, and selected zero-flux surfaces (left panel), visualization of electron localization function (ELF, center panel) and reduced density gradient (RDG, right panel) analyses for non-covalent interactions Br31···Br15, Se1···Br15, Se16···Br15, H13···Br15, and H13···Br31 in the model supramolecular associate **8**. Bond critical points (3, –1) are shown in blue, nuclear critical points (3, –3) – in pale brown, ring critical points – in orange, bond paths are shown as pale brown lines, length units – Å, and the color scale for the ELF and RDG maps is presented in a.u. The numeration of atoms corresponds to their ordering in the attached xyz-files for model supramolecular associates (Supplementary materials).

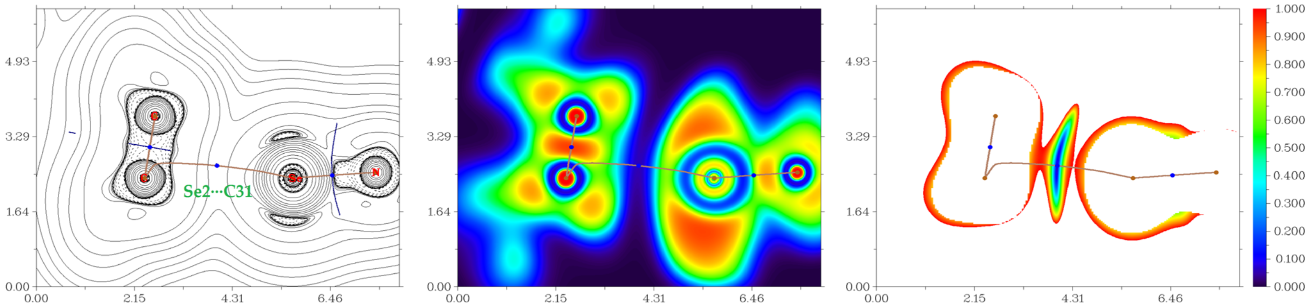

Figure S4. Contour line diagram of the Laplacian of electron density distribution ∇²ρ(**r**), bond paths, and selected zero-flux surfaces (left panel), visualization of electron localization function (ELF, center panel) and reduced density gradient (RDG, right panel) analyses for non-covalent interactions Se2···C31 in the model supramolecular associate **9**. Bond critical points (3, –1) are shown in blue, nuclear critical points (3, –3) – in pale brown, bond paths are shown as pale brown lines, length units – Å, and the color scale for the ELF and RDG maps is presented in a.u. The numeration of atoms corresponds to their ordering in the attached xyz-files for model supramolecular associates (Supplementary materials).

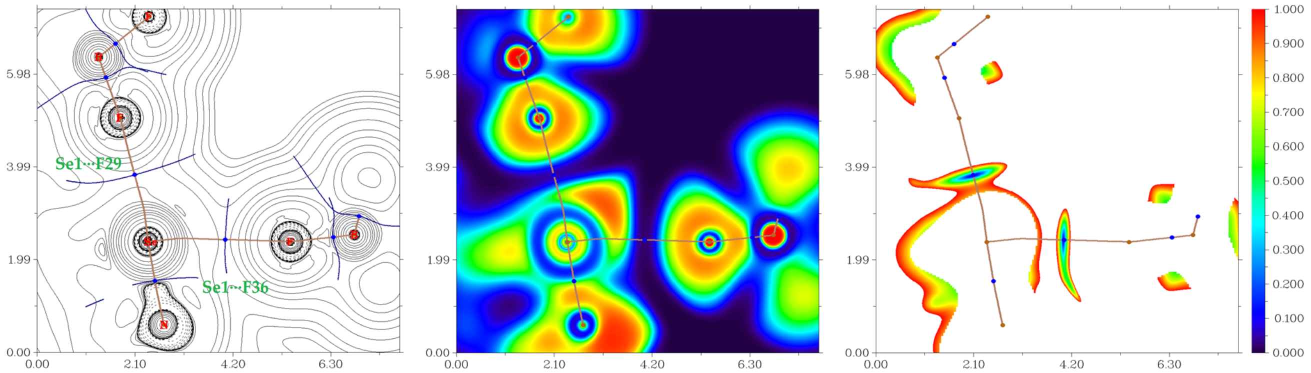

Figure S5. Contour line diagram of the Laplacian of electron density distribution $\nabla^2\rho(\mathbf{r})$, bond paths, and selected zero-flux surfaces (left panel), visualization of electron localization function (ELF, center panel) and reduced density gradient (RDG, right panel) analyses for non-covalent interactions Se1···F29 and Se1···F36 in the model supramolecular associate **10**. Bond critical points (3, –1) are shown in blue, nuclear critical points (3, –3) – in pale brown, bond paths are shown as pale brown lines, length units – Å, and the color scale for the ELF and RDG maps is presented in a.u. The numeration of atoms corresponds to their ordering in the attached xyz-files for model supramolecular associates (Supplementary materials).

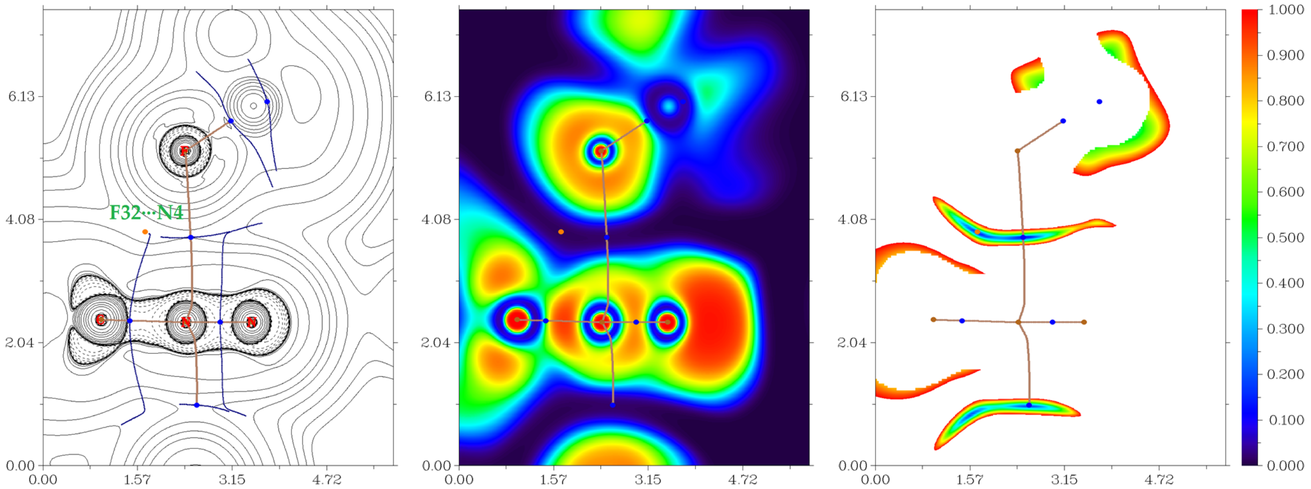

Figure S6. Contour line diagram of the Laplacian of electron density distribution $\nabla^2\rho(\mathbf{r})$, bond paths, and selected zero-flux surfaces (left panel), visualization of electron localization function (ELF, center panel) and reduced density gradient (RDG, right panel) analyses for non-covalent interactions F32···N4 in the model supramolecular associate **10**. Bond critical points (3, –1) are shown in blue, nuclear critical points (3, –3) – in pale brown, ring critical points – in orange, bond paths are shown as pale brown lines, length units – Å, and the color scale for the ELF and RDG maps is presented in a.u. The numeration of atoms corresponds to their ordering in the attached xyz-files for model supramolecular associates (Supplementary materials).
